# Epidemiology of *Taenia saginata* taeniosis/cysticercosis: a systematic review of the distribution in West and Central Africa

**DOI:** 10.1186/s13071-019-3584-7

**Published:** 2019-06-27

**Authors:** Emilie Hendrickx, Lian F. Thomas, Pierre Dorny, Branko Bobić, Uffe Christian Braae, Brecht Devleesschauwer, Ramon M. Eichenberger, Sarah Gabriël, Anastasios Saratsis, Paul R. Torgerson, Lucy J. Robertson, Veronique Dermauw

**Affiliations:** 1 Department of Biomedical Sciences, Institute of Tropical Medicine, Antwerp, Belgium; 2 International Livestock Research Institute (ILRI), P.O. Box 30709, Nairobi, Kenya; 3 Institute for Infection & Global Health, University of Liverpool, Neston, UK; 4 Department of Virology, Parasitology and Immunology, Faculty of Veterinary Medicine, Ghent University, Merelbeke, Belgium; 5 Centre of Excellence for Food and Vector-borne Zoonoses, Institute for Medical Research, University of Belgrade, Dr Subotića 4, Belgrade, 11000 Serbia; 6 One Health Center for Zoonoses and Tropical Veterinary Medicine, Ross University School of Veterinary Medicine, P.O. Box 334, Basseterre, Saint Kitts and Nevis; 7 Department of Infectious Disease Epidemiology and Prevention, Statens Serum Institut, 2300 Copenhagen, Denmark; 8 Department of Veterinary Public Health and Food Safety, Faculty of Veterinary Medicine, Ghent University, Merelbeke, Belgium; 9 Department of Epidemiology and Public Health, Sciensano, Brussels, Belgium; 10 Institute of Parasitology, Vetsuisse Faculty, University of Zurich, Zurich, Switzerland; 11 Veterinary Research Institute, Hellenic Agricultural Organisation Demeter, Thermi, 57001 Greece; 12 Department of Food Safety and Infection Biology, Faculty of Veterinary Medicine, Norwegian University of Life Sciences, Oslo, Norway

**Keywords:** *Taenia saginata*, Cysticercosis, Cattle, Beef, Tapeworm, West Africa, Central Africa

## Abstract

**Background:**

The zoonotic tapeworm *Taenia saginata*, although causing only minor discomfort in humans, is responsible for considerable economic losses in the livestock sector due to condemnation or downgrading of infected beef carcasses. An overview of current knowledge on the distribution and prevalence of this parasite in West and Central Africa is lacking.

**Methods:**

We conducted a systematic review, collecting information on published and grey literature about *T. saginata* taeniosis and bovine cysticercosis from 27 countries/territories in West and Central Africa, published between January 1st, 1990 and December 31st, 2017.

**Results:**

The literature search retrieved 1672 records, of which 51 and 45 were retained for a qualitative and quantitative synthesis, respectively. Non-specified human taeniosis cases were described for Nigeria, Cameroon, Senegal, Burkina Faso, Democratic Republic Congo, Guinea, and Ivory Coast (seven out of 27 countries/territories), while *T. saginata* taeniosis specifically was only reported for Cameroon. Most prevalence estimates for taeniosis ranged between 0–11%, while three studies from Nigeria reported prevalence estimates ranging between 23–50%. None of the studies included molecular confirmation of the causative species. The presence of bovine cysticercosis was reported for Benin, Burkina Faso, Cameroon, Central African Republic, Chad, Democratic Republic Congo, Ghana, Guinea, Ivory Coast, Mali, Niger, Nigeria, Senegal, and Tristan da Cunha (14 out of 27 countries/territories). Prevalence estimates ranged between 0–29%.

**Conclusions:**

Our systematic review has revealed that human taeniosis and bovine cysticercosis are seriously understudied in West and Central Africa. The high prevalence estimates of both conditions suggest an active dissemination of this parasite in the region, calling for a concerted One Health action from public health, veterinary health and food surveillance sectors.

**Electronic supplementary material:**

The online version of this article (10.1186/s13071-019-3584-7) contains supplementary material, which is available to authorized users.

## Background

The tapeworm *Taenia saginata* is one of three *Taenia* species that infect humans as their definitive host, with bovines serving as the intermediate host. Humans acquire *T. saginata* infection after consuming undercooked beef containing viable cysticerci. The adult tapeworm resides in the small intestine, where it becomes patent within approximately ten weeks. At that moment, the strobila may have reached a length of up to three meters [1], and gravid proglottids can contain up to 100,000 taeniid eggs. These eggs are voided during and between defecation [2], and have the potential to survive for a long time without hatching. Eggs found in faecal material and eggs within soil have been documented to remain viable for up to 9.5 months [3]. Contaminated pastures, water and feed are a source of infection for cattle. Following ingestion, the early larval stages (oncospheres) hatch and the hexacanth larvae migrate, utilising the lymphatic and blood system, to the muscle tissue. Here the larvae mature into the metacestode stage, called cysticerci [4].

Unlike *Taenia solium*, for which humans can also act as a dead-end intermediate host leading to the debilitating and stigmatising disease neurocysticercosis, human *T. saginata* infections are restricted to the definitive (adult tapeworm) stage, which has a more limited public health burden. *Taenia saginata* taeniosis is generally asymptomatic or associated with mild abdominal discomfort, although more serious complications, including appendicitis, intestinal obstruction and gall bladder perforation have occasionally been documented [3]. Bovine cysticercosis, however, may result in substantial economic losses generated for the food industry because of meat condemnation, treatment processing costs and an overall reduction in the product value [5]. Moreover, the meat inspection process itself requires substantial (veterinary) public health sector investment and there are costs associated with treatment seeking behaviour, diagnostics, and treatment of human taeniosis cases [5, 6].

*Taenia saginata* is considered to have a global distribution, with higher prevalences in low-income regions where sanitation standards may be poor, and the meat inspectorate services are often poorly funded and understaffed.

In West and Central Africa, the cattle population amounts to 120 million heads [7]. While West Africa mainly consists of arid (and to lesser degree semi-arid and sub-humid) agro-ecological zones, Central Africa predominantly consists of humid zones (with some sub-humid zones as well) [8]. In the purely humid agro-ecological zones, cattle production is not considered an important economic activity due to the presence of diseases such as trypanosomiasis [8]. In the arid zones, pastoralism is the most commonly cattle production system, while the semi-arid and sub-humid zones in the area are characterized by mixed crop-livestock farming systems [8].

As a summary of existing knowledge on the occurrence of *T. saginata* taeniosis and bovine cysticercosis in the area is presently lacking, and as part of a coordinated effort to document the global distribution of *T. saginata* [9–14], we undertook a systematic review of the occurrence of this parasite in West and Central Africa.

## Methods

### Search strategy

We conducted a systematic review aiming to gather current knowledge on the occurrence, prevalence and geographical distribution of human taeniosis and bovine cysticercosis in West and Central Africa, published between January 1st, 1990 and December 31st, 2017. A complete study protocol is available in Additional file 1: Text S1. In the context of this study, West and Central Africa was defined as the area covering the following 27 countries/territories: Ascension, Benin, Burkina Faso, Cameroon, Cape Verde, Central African Republic, Chad, Republic of the Congo, Democratic Republic of the Congo (DR Congo), Equatorial Guinea, Gabon, The Gambia, Ghana, Guinea, Guinea-Bissau, Ivory Coast, Liberia, Mali, Mauritania, Niger, Nigeria, Saint Helena, Sao Tome and Principe, Senegal, Sierra Leone, Togo and Tristan da Cunha. Although Angola is classified as being part of Central Africa, it is also classified as being part of southern Africa, and data from this country were included in an equivalent systematic review of southern and eastern Africa [14].

The international scientific databases Web of Science (http://ipscience.thomsonreuters.com/product/web-of-science/) and PubMed (http://www.ncbi.nlm.nih.gov/pubmed) were searched using the following combination of key words: (cysticerc* OR cisticerc* OR “C. bovis” OR taenia* OR tenia* OR saginata OR taeniosis OR teniosis OR taeniasis OR ténia OR taeniid OR cysticerque) AND (Ascension OR Benin OR “Burkina Faso” OR Cameroon OR “Cape Verde” OR “Central African Republic” OR Chad OR Congo-Brazzaville OR DRC OR Congo OR “Cote d’Ivoire” OR “Equatorial Guinea” OR Gabon OR Gambia OR Ghana OR Guinea OR Guinée OR Guinea-Bissau OR Liberia OR Mali OR Mauritania OR Niger OR Nigeria OR “Saint Helena” OR Sao Tome OR Principe OR Senegal OR “Sierra Leone” OR Togo OR “Tristan da Cunha”). Moreover, databases for MSc/PhD theses and grey literature (Additional file 2: Text S2) were searched using the same search phrase. Furthermore, the OIE databases “Help with World Animal Disease Status” (“Handistatus”, 1996–2004) [15] and “World Animal Health Information System” (“WAHIS”, 2005) [16] were consulted to extract data on bovine cysticercosis for the study area. Finally, reference lists of relevant reviews were screened for additional records.

### Selection criteria

The PRISMA guidelines were followed for reporting the review (Additional file 3: Table S1). Briefly, duplicate records were removed after compiling results from the different searches, followed by screening of titles and abstracts for relevance. Then, full text articles were evaluated using the following exclusion criteria: (i) studies concerning a parasite different from *T. saginata*; (ii) studies conducted outside the study area; (iii) studies published outside the study period; (iv) studies reporting results outside the scope of our review question (e.g. review, experiment, intervention); and (v) duplicated data. No language restrictions were implemented.

### Data extraction and compilation

Data from included records were extracted. Where records reported both the numerator and denominator of the study sample, respectively, prevalence and 95% Wilson score confidence intervals (CI) were calculated. All calculations were conducted in R, version 3.5.2.

## Results

### Search results

In total, 1670 records were retrieved, including 1655 through database searching, while 17 additional records were identified, including 15 retrieved through reference list screening, and the OIE databases Handistatus [15] and WAHIS [16]. After the removal of duplicates, out of 1237 remaining records, 1235 underwent title and abstract screening (i.e. the abstract was unavailable for 2 records). Subsequently, full texts of 87 articles were assessed for eligibility, of which 51 articles were retained for the qualitative synthesis (45 journal articles, 3 conference abstracts, 2 databases, 1 letter to the editor), of which 45 were included in the quantitative synthesis (Additional file 4: Figure S1).

### Human taeniosis

**Fig. 1:**
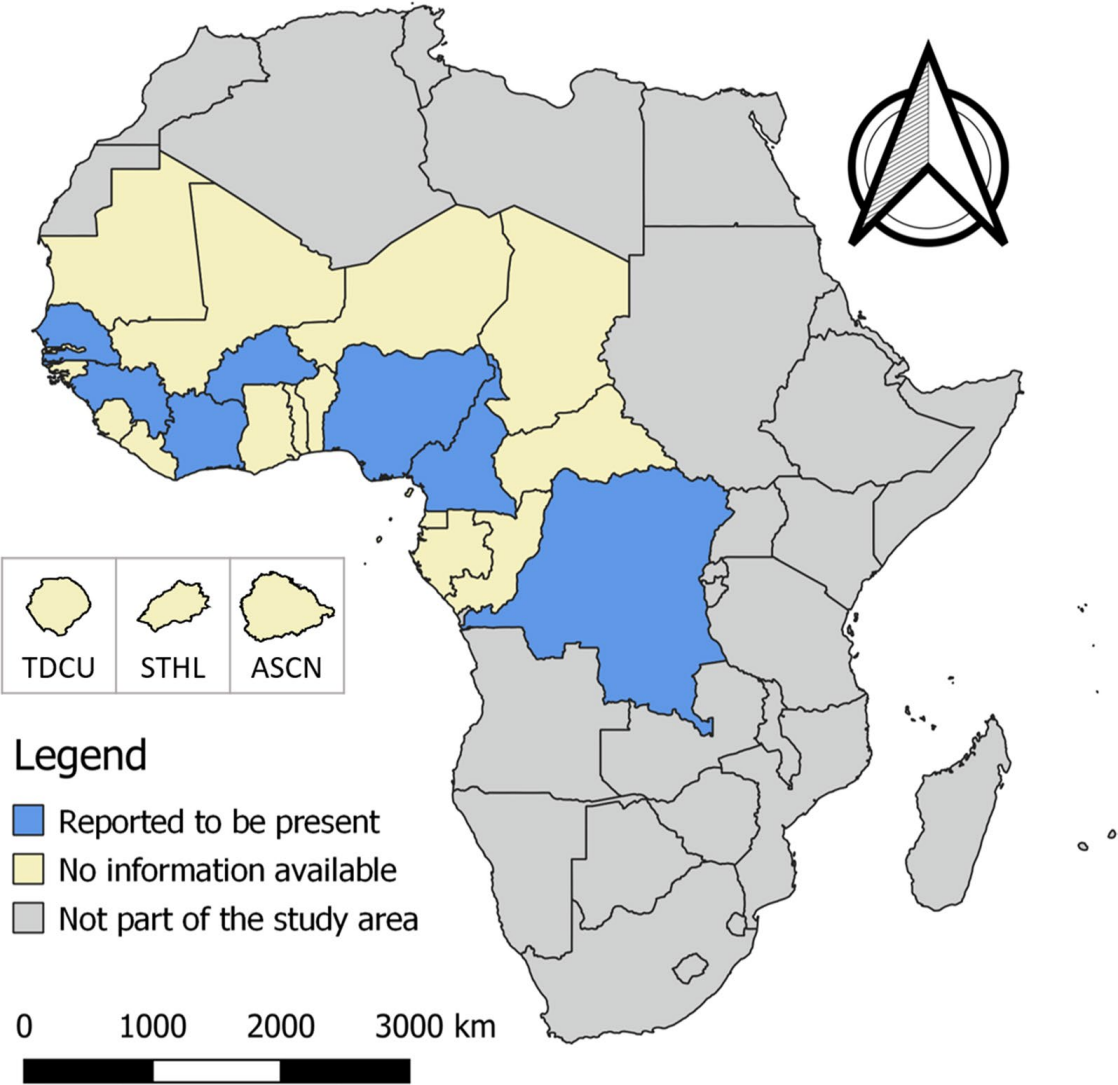
Human taeniosis in West and Central Africa. The islands Tristan da Cunha (TDCA), Saint Helena (STHL) and Ascension (ASCN) are magnified (i.e. they are not shown according to the given scale) to improve presentation

A total of 45 records described human taeniosis cases, of which 39 were included in the quantitative synthesis (Table 1). Out of 45 records, 35 describe results from Nigeria, 3 from Cameroon, 3 from Senegal, 1 from Burkina Faso, 1 from DR Congo, 1 from Guinea and 1 from Ivory Coast (Fig. 1). No data were available for the other countries in the study area.

**Table 1:** Reported occurrence of taeniosis in West and Central Africa

Country	Study period	*n*	*n*+	%	95% CI	Species	Groups studied	Reference
Burkina Faso	na	1587	na	2.1	na	N	Community volunteers	[39]
Cameroon	08/1999–04/2000	3109	1	0.03	0.006–0.18	Y^a^	Community volunteers	[35]
Cameroon	03/2012–07/2012	396	na	0.25	na	N	Patients consulting hospital	[40]
Cameroon	na	163	2	1.2	0.3–4.4	N	Pre-school-age children	[41]
DR Congo	03/04/2014–07/06/2018	602	40	6.6	4.9–8.9	Y^b^	School children (6–20 years)	[42]
Guinea	04/1995–06/1995	800	na	3.8	na	N	Children (10–14 years)	[43]
Nigeria	01/02/1997–31/01/1998	816	8	1.0	0.50–1.9	N	Pregnant women	[44]
Nigeria	07/02/1998–31/12/1998	129	3	2.3	0.8–6.6	Y^b^	Patients with complaints of upper abdominal pain, tenderness and indigestion	[45]
Nigeria	03/2000–09/2000	500	16	3.2	2.0–5.1	N	Schoolchildren	[46]
Nigeria	1/11/2003–30/1/2004	60	2	3.3	0.9–11.4	Y^b^	Children (2–6 years) attending clinic	[47]
Nigeria	11/2004–02/2005	232	23	9.9	6.7–14.4	N	Primary schoolchildren	[48]
Nigeria	01/2005–05/2005	309	5	1.6	[0.7; 3.7]	N	Schoolchildren	[49]
Nigeria	06/2005–11/2006	1059	102	9.6	8.0–11.6	N	Schoolchildren	[50]
Nigeria	10/2005–03/2006	119	1	0.8	0.2–4.6	N	Outpatients health institutions	[51]
Nigeria	1/2006–09/2006	73	24	32.9	23.2–44.3	N	Rural schoolchildren	[17]
Nigeria	1/2006–09/2006	171	19	11.1	7.2–16.7	N	Suburban schoolchildren	[17]
Nigeria	01/2006–09/2006	283	na	23.0	na	N	Primary schoolchildren	[19]
Nigeria	06/2006–11/2006	818	4	0.5	0.2–1.3	N	Hospital patients	[52]
Nigeria	01/2007–03/2007	250	1	0.4	0.07–2.2	N	Schoolchildren	[53]
Nigeria	07/2007–08/2007	100	13	13.0	7.8–21.0	N	Sampling in hostels (faeces sampling in toilets, not from individual participants)	[54]
Nigeria	08/2007–08/2009	500	0	0	0–0.8	N	HIV-negative enrolled via HIV outreach programme (in houses and offices)	[18]
Nigeria	08/2007–08/2009	2000	4	0.2	0.08–0.5	N	HIV-positive patients attending clinic	[18]
Nigeria	01/2008–08/2008	50	4	8	3.2–18.8	N	Villagers	[32]
Nigeria	05/2009–07/2009	122	2	1.6	0.5–5.8	N	Abattoir workers	[55]
Nigeria	05/2009–07/2009	98	na	0	na	N	Control populations	[55]
Nigeria	08/2010–12/2010	600	19	3.2	2.0–4.9	N	Schoolchildren	[56]
Nigeria	01/2011–12/2011	3826	na	0.89	na	N	Primary schoolchildren	[57]
Nigeria	06/2011–11/2011	220	na	5	na	N	Schoolchildren aged 1–15 years	[58]
Nigeria	06/2012–12/2012	116	10	8.6	4.7–15.1	Y^b^	Food vendors	[59]
Nigeria	09/2012–01/2013	717	5	0.7	0.3–1.6	N	Samples from polio surveillance programme	[60]
Nigeria	02/2014–06/2014	167	84	50.3	42.8–57.8	N	Pre-school-age children	[20]
Nigeria	07/2014–11/2014	112	3	2.7	0.9–7.6	N	Community volunteers	[61]
Nigeria	na	750	16	2.1	1.3–3.4	N	Women (child up to senior)	[62]
Nigeria	na	471	1	0.2	0.04–1.2	N	Primary schoolchildren	[63]
Nigeria	na	96	1	1	0.2–5.7	N	HIV patients	[64]
Nigeria	na	168	8	4.8	2.4–9.1	N	Food vendors	[65]
Nigeria	na	296	11	3.7	2.1–6.5	N	Villagers	[66]
Nigeria	na	162	2	1.2	0.3–4.4	N	Primary schoolchildren	[67]
Nigeria	na	400	12	3.0	1.7–5.2	N	Primary schoolchildren	[68]
Nigeria	na	416	45	10.8	8.2–14.2	N	Nursery and primary school children	[69]
Senegal	04/1997	400	na	3.5	na	N	Schoolchildren	[70]
Senegal	2004	na	na	4.6	na	N	Routine analyses at parasitology laboratory	[71]
Senegal	2005	na	na	5.6	na	N	Routine analyses at parasitology laboratory	[71]
Senegal	2006	na	na	4.0	na	N	Routine analyses at parasitology laboratory	[71]
Senegal	2007	na	na	7.0	na	N	Routine analyses at parasitology laboratory	[71]
Senegal	2008	na	na	5.5	na	N	Routine analyses at parasitology laboratory	[71]
Senegal	2009	na	na	4.1	na	N	Routine analyses at parasitology laboratory	[71]

^a^Species identification based on expelled worm

^b^Reported as *Taenia saginata*, yet unclear from methodology how species identification was done

*Abbreviations*: *n*, number of individuals tested; *n*+, number of positive individuals; CI, confidence interval; na, not available; Y, yes; N, no

All studies included in the quantitative synthesis used plain stool microscopy as a diagnostic tool, and prevalence estimates for taeniosis ranged between 0–11% (0% in suburban schoolchildren [17] and 11% in community residents [18] both in Nigeria). Three other studies performed in Nigeria; however, they reported much higher prevalence estimates: 23% in primary schoolchildren [19], 33% in rural schoolchildren [17] and even 50% in pre-school-age children (aged between 0 and 71 months) [20]. In four other studies, excluded from the quantitative synthesis, the presence of *T. saginata* taeniosis was described for Nigeria, Ivory Coast and Senegal, without prevalence estimates [21–24]. A further two case reports were also excluded from the qualitative synthesis: the first case described the presence of Meckel’s diverticulitis due to *T. saginata* taeniosis in a 6-year-old girl in Nigeria [25], and the other a case of *T. saginata* taeniosis in a 33-year-old male presumably infected in Ivory Coast but diagnosed in Spain [26]. Only five studies reported the specific presence of *T. saginata* taeniosis; none of the studies mentioned morphological identification or molecular confirmation of the causative species, although one study conducted in Cameroon mentioned that species identification was done based on the expelled worm.

### Bovine cysticercosis

Only 4 journal articles in addition to the 2 OIE databases described the presence of bovine cysticercosis in the study area, 3 of which were included in the quantitative synthesis (Table 2). The journal articles (1 described data for DR Congo, 3 for Nigeria) reported prevalence estimates based on abattoir surveys (i.e. meat inspection) between 0–29.0% (0% [27], 29% [28], both in Nigeria). One article, which was excluded from the quantitative synthesis, described the presence of bovine cysticercosis in Nigeria, without prevalence estimates [23].

**Table 2:** Reported occurrence of bovine cysticercosis in West and Central Africa: reports based on meat inspection

Country	Study period	Data source	*n*	*n*+	%	95% CI	Reference
Congo	06/1986–06/1987	Veterinary inspection records	3914	na	9.6	na	[72]
Congo	07/1986–10/1988	Veterinary inspection records	333	na	10.5	na	[72]
Congo	07/1986–10/1988	Veterinary inspection records	284	na	12.7	na	[72]
Congo	na	Veterinary inspection records	73	na	1.4	na	[72]
Congo	na	Veterinary inspection records	47	na	4.3	na	[72]
Congo	na	Veterinary inspection records	35	na	14.3	na	[72]
Nigeria	1985–1986	Veterinary inspection records	1221	0	0	0–0.3	[27]
Nigeria	1985–1986	Retail market inspection by investigators	358	27	7.5	5.2–10.8	[27]
Nigeria	11/1999–04/2002	Carcass inspection by investigators	5560	1,610	29.0	27.8–30.2	[28]^a^
Nigeria	11/1999–04/2002	Carcass inspection by investigators	20,240	5,140	25.4	24.8–26.0	[28]^b^
Nigeria	2005–2007	Veterinary inspection records	641,224	805	0.13	0.12–0.13	[73]

^a^Local breeds in rural areas

^b^Exotic breeds in urban areas

*Abbreviations*: *n*, number of individuals tested; *n*+, number of positive individuals; CI, confidence interval; na, not available

**Fig. 2:**
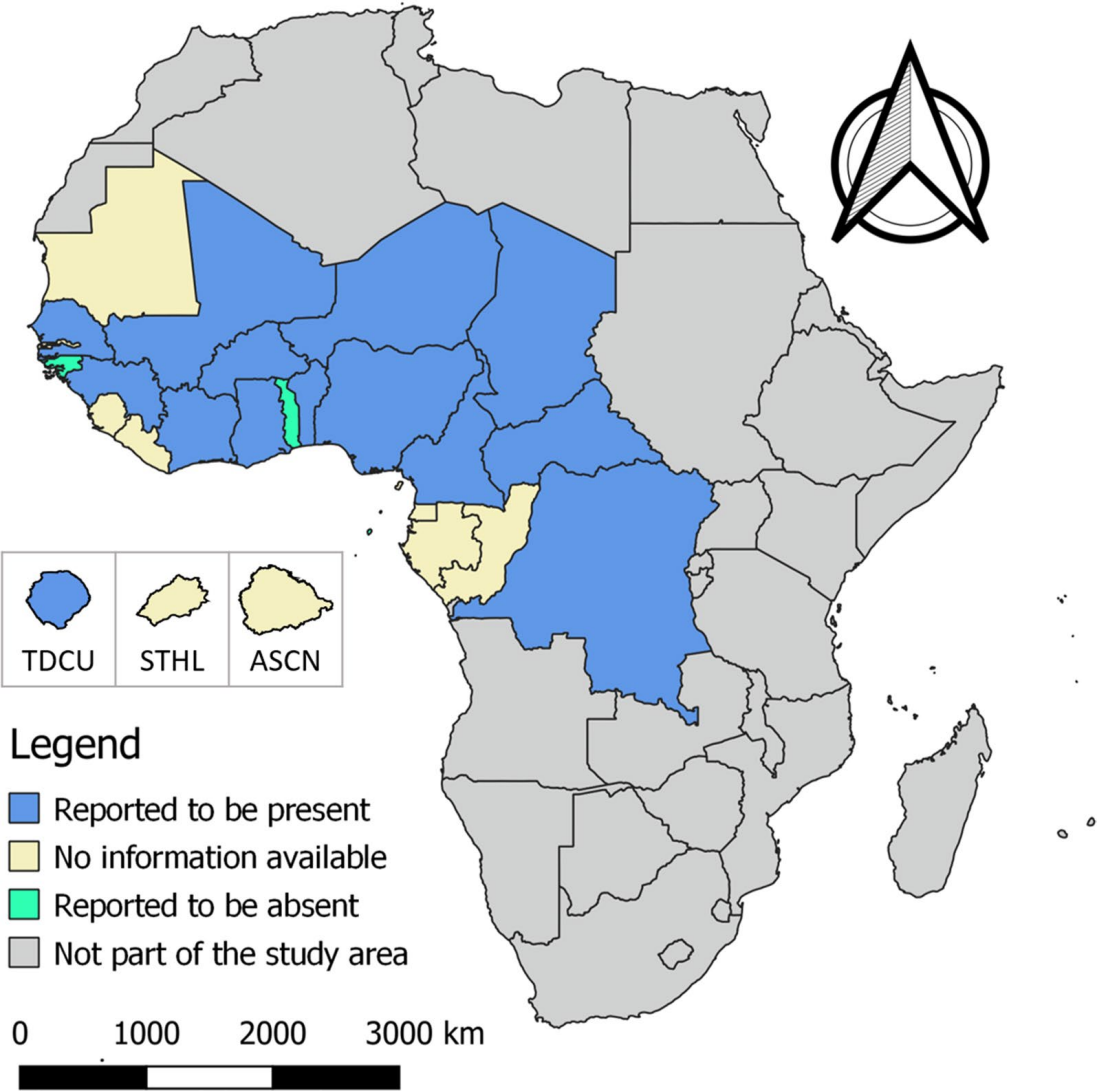
Bovine cysticercosis in West and Central Africa. The islands Tristan da Cunha (TDCA), Saint Helena (STHL) and Ascension (ASCN) are magnified (i.e. they are not shown according to the given scale) to improve presentation

In contrast with the journal articles, the OIE databases reported the (past) presence of bovine cysticercosis in a larger part of the study area (Table 3). Overall, bovine cysticercosis was reported throughout the study area, except for Guinea-Bissau, Sao Tomé and Principe, and Togo, where it was declared to be absent [15, 16] (Fig. 2). No data were available for Ascension, Cape Verde, the Republic of Congo, Equatorial Guinea, Gabon, Gambia, Liberia, Mauritania, Saint Helena or Sierra Leone.

**Table 3:** OIE data on occurrence of bovine cysticercosis in West and Central Africa (1996–2005) [15, 16]

Country/territory	1996	1997	1998	1999	2000	2001	2002	2003	2004	2005
Ascension										
Benin								+	79	
Burkina Faso	+	+	+							+
Cameroon		+	+	+	+	+	+	21	23	+
Cape Verde										
Central African Republic	+					+	+		+	+
Chad							+			
DR Congo							+			+
Republic of the Congo										
Equatorial Guinea										
Gabon										
Gambia										
Ghana		+								-
Guinea	+									
Guinea-Bissau							-	-	-	
Ivory Coast	+	+	+	+	+	+	+	+	+	
Liberia										
Mali	+									-
Mauritania										
Niger	+	+				+				
Nigeria				+		+	+	153	34	-
Saint Helena										
Sao Tome and Principe					-	-	-	-	-	
Senegal	92	+	199	125	+	+				
Sierra Leone										
Togo										-
Tristan da Cunha		71			1	+		1		

Blank cells indicate the data were unavailable

*Abbreviations*: CI, confidence interval; +, occurrence of the disease; -, absence of the disease

## Discussion

Our aim was to gather current knowledge on human taeniosis and bovine cysticercosis in West and Central Africa. Overall, human taeniosis was reported in seven out of 27 countries/territories, while bovine cysticercosis was reported in 14 out of 27 countries/territories. This systematic review has revealed that human *T. saginata* taeniosis and bovine cysticercosis are seriously understudied in this region. While the study area consists of 27 countries and territories, the presence of human taeniosis and bovine cysticercosis were only described for 7 and 17 countries, respectively. For the remaining regions no data were reported. These findings are in contrast to eastern and southern Africa which have similar lifestyles yet a remarkably higher coverage [12]. This difference might be partially explained by the presence of a large French speaking population in West and Central Africa, and therefore potentially published research in journals which were not detected completely by our search strategy, although we had included French search terms and some articles in French were included in the present study. Another explanation might be a lower awareness and interest in the topic, as the cattle population in this area is somewhat lower as compared to eastern and southern Africa, i.e. 120 million heads [7] as opposed to 184 million heads [7].

Overall, the reported prevalence estimates of taeniosis were in line with those reported in eastern and southern Africa [12] and in the Americas [13], but higher than those reported in western and eastern Europe [9, 10]. In contrast to certain areas in eastern and southern Africa where consumption of raw beef is a culinary habit [12], traditional dishes in West and Central Africa include mainly stews with typically extended cooking times [29, 30], thereby decreasing the risk of exposure to viable *T. saginata* cysts. Three articles, however, reported very high taeniosis prevalence estimates, with 23% in primary schoolchildren [19], 33% in rural schoolchildren [17] and even 50% in pre-school-age children [20]. According to Adeniran et al. [20], Nigerian pre-school-age children are often fed undercooked meals for adults, including beef, in order to facilitate the transition from breastmilk to solid food. Should this high prevalence estimate be confirmed, such practices should be investigated and be the subject of close attention and education on the potential dangers associated with consumption of inadequately cooked food. In all taeniosis cases, species identification is pivotal to avoid the potential ingestion of *T. solium* eggs by the tapeworm carrier, and relatives and acquaintances, representing a risk of neurocysticercosis. *Taenia solium* is endemic in many of the included countries or territories [31–36]. Most studies, however, reported non-specified taeniosis, and for those specifically mentioning *T. saginata* taeniosis only one described the applied method. None of the other studies reported the use of specific morphological methods or molecular tools allowing for the identification of the causative *Taenia* sp. [9]. Hence, we cannot rule out that a certain proportion of taeniosis cases summarized in this review are due to *T. solium* instead of *T. saginata*.

A wide fluctuation in prevalence estimates, ranging between 10–30% for most studies [37] was observed for bovine cysticercosis, which is comparable to the estimates for eastern and southern Africa [12]. In many countries, meat inspection is not done systematically, especially in the case of backyard slaughtering in rural areas where meat inspection may not be available. Moreover, a correct estimation of the prevalence through meat inspection is hampered by its low sensitivity, which is estimated to lie below 16% [38]. This was confirmed by one study from Nigeria describing the prevalence of bovine cysticercosis in carcasses having passed the regular meat inspection at the abattoir. While the regular veterinary inspection declared the carcasses to be free from cysticercosis, investigators found a prevalence of 7.5% in carcasses originating from the same abattoir, sold at retail markets [27]. Overall, prevalence estimates for taeniosis of up to 50%, and for bovine cysticercosis of up to 30%, indicate the continued transmission of this parasite between cattle and humans. Despite the limited pathology caused by *T. saginata*, bovine cysticercosis has the potential to cause a high economic cost due to condemnation of infected carcasses. To interrupt transmission, stringent meat inspection procedures should be applied and improvements implemented in the sanitation and management of human sewage. The public should also be educated regarding general food safety measures such as thorough cooking of meat products, which also reduces the risk of infection with other microbiological hazards associated with meat products, such as pathogenic *Escherichia coli*, *Salmonella* spp., *Mycobacterium bovis* and *Campylobacter* spp.

## Conclusions

Based on the findings of our systematic review, both human taeniosis and bovine cysticercosis are understudied in West and Central Africa. Included articles reported high prevalence estimates for both conditions, pointing to a continued transmission of *T. saginata* in the region. A One Health approach is needed to protect the general public from acquiring tapeworm infection.

## Additional files

**Additional file 1: Text S1.** Search protocol.

**Additional file 2: Text S2.** Databases used.

**Additional file 3: Table S1.** PRISMA checklist.

**Additional file 4: Figure S1.** PRISMA flow diagram.

## Data Availability

All references found eligible in our literature review are included in the article.

## Abbreviations

CI: confidence interval
DR Congo: Democratic Republic of the Congo
OIE: World Organisation for Animal Health/Office International des Epizooties
PRISMA: Preferred Reporting Items for Systematic Reviews and Meta-Analyses
WAHIS: World Animal Health Information System
