# Supplementary material for: Epidemiology of Taenia saginata taeniosis/cysticercosis: a systematic review of the distribution in West and Central Africa
Source: Parasit Vectors. 2019 Jun 27;12:324. doi: 10.1186/s13071-019-3584-7 (PMC6598244; doi:10.1186/s13071-019-3584-7)
Supplement: Supplementary file 1 — Additional file 1: Text S1. Search protocol. [file 13071_2019_3584_MOESM1_ESM.docx]

**Epidemiology of *Taenia* *saginata* taeniosis/cysticercosis: a systematic review of the distribution in West and Central Africa:**

**Additional file 1: Text S1. Search protocol**

**Aim:** To synthesis the current evidence on presence/absence and prevalence (where reported) of *T. saginata* (taeniosis/cysticercosis) in West & Central Africa

**Questions to answer:**

Which countries have reported the presence of *T. saginata* in cattle and/or humans since 1990?

For which countries are prevalence data available and what is the quality of that data?

Are specific geographical locations available for these data?

**Methods:**

The review will be conducted in line with the PRIMSA statement 2009 (<http://www.bmj.com/content/339/bmj.b2700#alternate>) and will include each item on the PRIMSA checklist <http://www.prisma-statement.org>.

Articles will be selected for inclusion into the systematic review through the identification of all potentially relevant citations through the search strategy. The citations within identified articles will also be included in the screening process. Duplicates will be excluded, followed by screening of titles and abstracts with articles excluded if they do not explicitly report occurrence or prevalence of *T. saginata*. Full text articles will then be screened for exclusion criteria with those remaining were included in the review.

Full text articles will then be read, and relevant data extracted and summarized into a Microsoft Excel spreadsheet.

**Databases: See Supplementary Material 2**

**Search term (Pubmed):** (cysticerc* OR cisticerc* OR “C. bovis” OR taenia* OR tenia* OR saginata OR taeniosis OR teniosis OR taeniasis OR ténia OR taeniid OR cysticerque) AND (Ascension OR Benin OR "Burkina Faso" OR Cameroon OR "Cape Verde" OR "Central African Republic" OR Chad OR Congo-Brazzaville OR DRC OR Congo OR "Cote d’Ivoire" OR "Equatorial Guinea" OR Gabon OR Gambia OR Ghana OR Guinea OR Guinée OR Guinea-Bissau OR Liberia OR Mali OR Mauritania OR Niger OR Nigeria OR "Saint Helena" OR Sao Tome OR Principe OR Senegal OR "Sierra Leone" OR Togo OR "Tristan da Cunha")

**Inclusion/exclusion:**

- **Exclusion criteria:**
  - studies concerning a different parasite than *T. saginata*
  - studies reporting data from outside study area
  - studies reporting/using data older than 1990 or published after December 31^st^ 2017
  - studies reporting results out of the scope of the review questions
  - duplicated data
- **Languages:** All
- **Year data collection:** 1^st^ January 1990 – 31^st^ December 2017
- **Geographical range:** All countries/territories within or islands in proximity of western & central Africa
