# Supplementary material for: Epidemiology of Taenia saginata taeniosis/cysticercosis: a systematic review of the distribution in West and Central Africa
Source: Parasit Vectors. 2019 Jun 27;12:324. doi: 10.1186/s13071-019-3584-7 (PMC6598244; doi:10.1186/s13071-019-3584-7)
Supplement: Supplementary file 2 — Additional file 2: Text S2. Databases used. [file 13071_2019_3584_MOESM2_ESM.docx]

**Epidemiology of *Taenia* *saginata* taeniosis/cysticercosis: a systematic review of the distribution in West and Central Africa:**

**Additional file 2. Text S2. Databases used**

**Databases searched using keywords from the search phrase mentioned in the Materials and Methods section:**

- Pubmed (<https://www.ncbi.nlm.nih.gov/pubmed/>)
- Web of Science (<http://ipscience.thomsonreuters.com/product/web-of-science/>),
- OpenGrey (<http://www.opengrey.eu/>),
- OAIster (<http://oaister.worldcat.org/>),
- CABDirect (<http://www.cabdirect.org/>),
- Registry of Open Access repositories (<http://roar.eprints.org/cgi/search/advanced>),
- OpenDOAR (<http://www.opendoar.org>),
- USDA National Agriculture Library (<http://agricola.nal.usda.gov/>),
- Japan; J-stage (<https://www.jstage.jst.go.jp/browse>),
- Russia: District public health departments (<http://rospotrebnadzor.ru/>),
- Journals online, Asia (<http://asiajol.info/>),
- Journals online, Africa (<http://ajol.info/>),
- Journals online, Central America (<http://lamjol.info/>),
- WHO IRIS: (<http://apps.who.int/iris/>),
- Index Medicus for South-East Asian Region: (<http://imsear.hellis.org/>),
- African Index Medicus (AIM): (<http://indexmedicus.afro.who.int/>),
- Index Medicus for the Eastern Mediterranean Region (IMEMR) (<http://www.emro.who.int/information-resources/imemr-database/>),
- Western Pacific Region Index Medicus (WPRIM): (<http://www.wprim.org/>),
- China National Knowledge Infrastructure: (<http://en.cnki.com.cn/>),
- Networked Digital Library of Theses and Dissertations (NDLTD): (<http://www.ndltd.org/>),
- British Library E-thesis online service: (<http://ethos.bl.uk/Home.do;jsessionid=ADD34566923B6F193366A61D8D009239>),
- Swedish University Dissertations: (<http://www.dissertations.se/>),
- Spanish PhD Thesis: (<https://www.educacion.gob.es/teseo/irGestionarConsulta.do;jsessionid=2DA8162C9E0FF872824BA69A64FB784A>),
- French Thesis Online: (<http://www.theses.fr/>),
- TesiOnline (Italy): (<http://www.tesionline.com/intl/index.jsp>),
- German National Library: (<http://www.dnb.de/EN/Home/home_node.html>),
- Thesis Canada: (<http://amicus.collectionscanada.gc.ca/thesescanada-bin/Main/AdvSearch?coll=18&l=0&v=1>),
- Australasia Digital Theses Program: (<http://adt.caul.edu.au/>),
- Center for Research Libraries: (<http://catalog.crl.edu/search~S4>).
