# Supplementary material for: Epidemiology of Taenia saginata taeniosis/cysticercosis: a systematic review of the distribution in West and Central Africa
Source: Parasit Vectors. 2019 Jun 27;12:324. doi: 10.1186/s13071-019-3584-7 (PMC6598244; doi:10.1186/s13071-019-3584-7)
Supplement: Supplementary file 4 — Additional file 4: Figure S1. PRISMA flow diagram. [file 13071_2019_3584_MOESM4_ESM.doc]

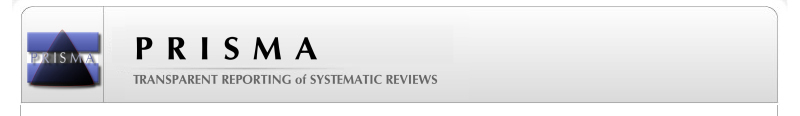
**Additional file 4: Figure S1. PRISMA 2009 Flow Diagram**

**Screening**

**Included**

**Eligibility**

**Identification**

Records identified through database searching
(n = 1655)

Additional records identified through other sources
(n = 17)

Records after duplicates removed
(n = 1220+17)

Records screened
(n = 1218+17)

Records excluded
(n = 1135)

Full-text articles assessed for eligibility
(n = 71+16)

Full-text articles excluded, with reasons
(n = 36): on a different parasite (n = 14), out of scope of research question (n = 19), out of study area (n = 2), duplicate record (n = 1)

Studies included in qualitative synthesis
(n = 36+15)

Studies included in quantitative synthesis
(n = 30+15)
